# Serum pepsinogen levels in different regions of China and its influencing factors: a multicenter cross-sectional study

**DOI:** 10.1186/s12876-021-01794-6

**Published:** 2021-06-12

**Authors:** Yuling Tong, Hongguang Wang, Yi Zhao, Xueqiang He, Hongwei Xu, Hong Li, Ping Shuai, Lirong Gong, Hongbo Wu, Hongzhi Xu, Yinhu Luo, Dong Wang, Shizhu Liu, Zhenya Song

**Affiliations:** 1 Department of General Practice/Health Management Center, The Second Affiliated Hospital of Zhejiang University, School of Medicine, No. 88 Jiefang Road, Hangzhou, China; 2 Department of Gastroenterology, Jilin City People’s Hospital, Jilin, China; 3 Department of Gastroenterology, No. 924 Hospital of the People’s Liberation Army of China, Guilin, China; 4 Department of Gastroenterology, Kunshan Hospital of Traditional Chinese Medicine, Kunshan, China; 5 Department of Health Medicine, Chinese PLA General Hospital, Beijing, China; 6 Health Management Center, Sichuan Provincial People’s Hospital, University of Electronic Science and Technology of China, Chengdu, China; 7 Department of Gastroenterology, The First Hospital Affiliated to AMU (Southwest Hospital), Chongqing, China; 8 Department of Gastroenterology, Zhongshan Hospital Affiliated to Xiamen University, Xiamen, China; 9 Department of Gastroenterology, Jingzhou Hospital of Traditional Chinese Medicine, Jingzhou, China; 10 Department of Gastroenterology, Shanghai Changhai Hospital, Shanghai, China

**Keywords:** Serum pepsinogen, Different regions of China, Influencing factors, Baseline survey

## Abstract

**Background:**

The aim of this study is to investigate the difference of serum pepsinogen (PG) baseline levels in different regions of China and its influencing factors.

**Methods:**

From October 2016 to October 2018, asymptomatic health checkup people who underwent nasal endoscopy in nine health management centers in different regions of China were collected. Lifestyle questionnaires were conducted, and serum PG and gastroscopy were performed. The differences in PG levels in baseline population (OLGA-0 grade) were studied according to geographical subregions of China. SPSS software was used for statistical analysis.

**Results:**

1922 patients were included in the final analysis. Compared with the non-atrophy (OLGA-0) group, PGR levels in atrophy group (OLGA-I to IV) were significantly decreased with the atrophy degree (*p* < 0.05). A total of 1590 baseline people (OLGA-0) were included in the study, including 254 from South China, 574 from East China, 210 from Southwest China, 332 from Northeast China, and 220 from Central/Northern China. There were significant differences in baseline PGI levels among the five regions (*p* < 0.05). The PGII levels were also different among the five regions, except for Central/Northern versus Southern China. PGR (PGI/PGII ratio) levels in Southern China were higher than other four regions. Further studies were conducted on the related factors that might affect the baseline PG level, which was affected by nationality, dietary habits, smoking, *Helicobacter pylori* infection and other related factors.

**Conclusion:**

Influenced by many factors, the baseline PG levels are different in different regions of China. In the follow-up studies of PG cut-off value, different PG cut-off value based on region may be more effective in the screening of gastric cancer and precancerous lesions in China.

## Background

Serum Pepsinogen (PG) can reflect the morphological and functional status of gastric mucosa. Pepsinogen I (PGI) is a pointer to detect the cell function of gastric acid gland (fundus gland), and decreased when gastric acid secretion is decreased or gastric mucosal glands are atrophied. Pepsinogen II (PGII) is significantly correlated with gastric fundus mucosa lesions (compared with gastric antrum mucosa), and its elevation is correlated with gastric fundus duct atrophy, intestinal metaplasia or pseudopylori metaplasia, and dysplasia. Progressive reduction of PGR (PGI/PGII ratio) is associated with progressive gastric mucosal atrophy and gastric cancer [1]. Multiple studies have confirmed that serum PG is a potential serological marker for screening gastric cancer and atrophic gastritis [2, 3].

PG level is affected by many factors such as race, region, age, gender, height, weight, body surface area, smoking history, drinking history, *Helicobacter pylori* (*Hp*) and so on [4]. Multiple studies have found that the cutoff value of PG in the diagnosis of gastric atrophy is different in different countries. The Japanese standard, taking PGI ≤ 70 ng/ml and PGR ≤ 3 as the cut-off value, is most widely used at present, with the sensitivity of 66.7–84.6% and specificity of 73.5–87.1%, for the diagnosis of atrophic gastritiss [5–7]. A number of European studies suggested that the cut-off value for evaluating fundus atrophy was PGI ≤ 56 ng/ml (sensitivity: 61.9%, specificity: 94.8%), and PGR ≤ 5 (sensitivity: 75.0%, specificity: 91.0%) [8]. A study from South Korea suggested that PGI ≤ 70 ng/ml had a good sensitivity (72.4%), but a low specificity (20.2%) in the evaluation of atrophic gastritis, while the sensitivity and specificity of PGR ≤ 3 were 59.2–61.7% and 61.0% [9]. According to the results of our previous studies, the PGR cutoff value for diagnosis of atrophy and severe atrophy is around 4.28–9.08 [10], which is much higher than the standard of PGR < 3 in Japan. The same differences were found in many domestic studies [11, 12], which may be related to the different baseline levels of PG. China is a vast country, it is necessary to explore whether there are significant differences of the baseline PG levels among different regions. In view of this situation, we combined with several domestic health management centers to carry out this research. Gastroscopy and pathology were adopted as the gold standard to detect serum PG in asymptomatic health checkup people and to study the influencing factors of the baseline levels of PG.

## Methods

### Participants

This was a multicenter cross-sectional study of consecutive subjects who underwent regular health checkup from October 2016 to October 2018 at nine International Healthcare Centers in different regions of China, including Southern China (No. 924 Hospital of the People’s Liberation Army of China), Eastern China (the Second Affiliated Hospital of Zhejiang University College of Medicine, Zhongshan Hospital affiliated to Xiamen University, and Traditional Chinese Medicine Hospital of Kunshan), Southwest China (Sichuan Provincial People’s Hospital and the First Hospital affiliated to AMU [Southwest Hospital]), Northeast China (Jinlin People’s Hospital), and Central/Northern China (Chinese PLA General Hospital and Jingzhou Hospital of Traditional Chinese Medicine). All procedures were performed in accordance with the ethical standards of the responsible committee on human experimentation (institutional and national) and the Helsinki Declaration of 1964 and later versions. Informed consent was obtained from all patients prior to enrolment. The study was approved and authorized by the ethics committees of various participating hospitals (approval #2015-079 at the Second Affiliated Hospital of Zhejiang University College of Medicine, the leading site).

Inclusion criteria were: (1) intention to undergo gastroscopy during health checkup examination; (2) 25–75 years of age. Exclusion criteria were: (1) a history of gastric ulcer, gastric polyp, or GC; (2) a history of gastrectomy; (3) treatment with a proton pump inhibitor in the last month; (4) contraindications to gastroscopy; (5) a history of *Hp* eradication; or (6) incomplete data.

### Questionnaire survey

A self-reported questionnaire was used in the present study. It included baseline information (age, sex and nationality), living style (smoking [> 1 cigarette daily for more than 1 year; the number of cigarettes and duration of smoking were asked for smokers], alcohol consumption [any type of alcohol more than once weekly for more than 1 year; alcohol types and consumption frequency were asked for drinkers], eating habits (high-salt diet [salt > 10 g/day], green vegetables and fresh fruits [> three times per week]), and family history of GC among first-degree relatives.

### Tests

The participants underwent gastroscopy and pathological examination of the biopsies, serum PG test, 13C-urea breath test (Shenzhen Zhonghe Headway Bio-Sci & Tech Co., Ltd., China), and/or *Hp* serological current infection marker rapid test (MP Biomedicals, Santa Ana, CA, USA), all on the same day. All tests were performed according to the manufacturers’ instructions.

*Hp* infection was determined based on the 13C-urea breath test, *Hp* serological current infection marker rapid test, and pathological screening. Patients showing positive results for any of these three tests were considered to be *Hp*-positive. If all tests were negative, the patient was considered to be *Hp*-negative.

Fasting blood (5 ml) was collected from each subject and centrifuged for 10 min at ≥ 10,000*g*. Serum PG levels were assayed by the chemiluminescent microparticle immunoassay method with the Abbott ARCHITECT Pepsinogen I and II Reagent Kit (Abbott Laboratories Inc., Chicago, IL, USA).

Gastroscopy was performed by the double-blind method. Two biopsies were performed at the small curvatures of the gastric antrum and body, respectively. Additional biopsies were taken from the mucosal abnormalities. The biopsies were scored semi-quantitatively by two pathologists with > 10 years of experience, according to the updated Sydney classification system [13] and the OLGA (Operative Link on Gastritis Assessment) method, which combine the degree and range of gastric mucosa atrophy/intestinal metaplasia, which are internationally accepted and applied in the screening of GC and precancerous lesions [14]. In case of disagreement, the two pathologists discussed the data until consensus.

### Statistical analysis

Statistical analysis was performed with SPSS 20 (IBM Corp., Armonk, NY, USA). Continuous data were tested for normal distribution by the Kolmogorov–Smirnov test. Data with normal distribution were expressed as mean ± standard deviation (SD), while data with skewed distribution were presented as median (interquartile ranges) (IQR), and compared by ANOVA with post hoc Scheffe’s test. Categorical data were presented as frequency and percentage, and analyzed by the chi-square test and Bonferroni post hoc test. *p* < 0.05 was considered statistically significant.

## Results

A total of 2256 subjects were included in the study. Totally 316 patients were excluded due to incomplete data, four due to a history of gastrectomy, five due to proton pump inhibitor use in the recent one month, and nine due to a history of gastric ulcer or gastric polyp. Finally, 1922 patients were included in the final analysis. The participants were 52.3 ± 9.8 years old. The male to female ratio was 1.2:1 (1065/857). There were 1590 participants in the OLGA-0 group, 185 in the OLGA-I group, 88 in the OLGA-II group, 43 in the OLGA-III group, 6 in the OLGA-IV group, and 10 in the gastric cancer (GC) group. There were no gender differences among the five groups. The age and *Hp* infection rate increased gradually with the increase of atrophy degree (Table 1).

**Table 1:** Age, sex and *Hp* infection rate in different groups

Group	Total (n = 1922)	OLGA-0 (n = 1590)	OLGA-I (n = 185)	OLGA-II (n = 88)	OLGA-III (n = 43)	OLGA-IV (n = 6)	GC (n = 10)
Gender (male), n (%)	1065 (55.4)	866 (54.5)	109 (58.9)	57 (64.8)	25 (58.1)	3 (50.0)	5 (50.0)
Age (years)	52.3 ± 9.8	51.9 ± 9.9	54.99 ± 9.1*	53.49 ± 8.8	55.42 ± 9.9*	56.33 ± 8.2*	59.8 ± 15.0*
*Hp* infection rate, n (%)	757 (39.4)	583 (36.7)	86 (46.5)*	51 (60.0)*	27 (62.8)*	5 (83.3)*	5 (50)

*Compared with OLGA-0 group, *p* < 0.05

### Serum PG differences among the pathological groups

Serum PG levels were compared among the NAG (non-atrophy group, OLGA-0 group), MAG (mile-moderate atrophy group, OLGA I-II group), SAG (severe atrophy group, OLGA III-IV group), and GC groups. Compared with NAG group, PG levels in atrophy group (OLGA-I to IV) were significantly different (*p* < 0.05). In addition, compared with the NAG group, PGI levels and PGRs in the MAG and SAG groups were significantly lower (*p* < 0.05). Compared with the MAG group, the SAG group had significantly lower PGRs (*p* < 0.05). PGR levels in the GC group were significantly lower than those of the NAG and MAG groups (*p* < 0.05). The PG levels between the SAG and GC groups had no significant differences (*p* > 0.05) (Table 2).

**Table 2:** The PG levels in different pathologic groups

	Non-atrophy (OLGA-0) (n = 1590)	Atrophy (OLGA I-IV)			GC group (n = 10)
		Total (n = 322)	MAG (OLGA I-II) (n = 273)	SAG (OLGA III-IV) (n = 49)	
PGI (ng/ml)	90.4 (85.2)	67.8 (71.0)*	69.5 (76.1)*	60.8 (53.8)*	64.9 (77.5)
PGII (ng/ml)	8.3 (11.7)	9.2 (9.7)*	8.7 (9.4)	11.9 (12.3)*	11.1 (19.7)
PGR	10.1 (16.0)	7.7 (5.2)*	7.9 (5.4)*^#^	6.3 (4.5)*^#^	4.5 (10.2)*^#^

*MAG* mild-moderate atrophy group, *SAG* severe atrophy group

*p < 0.05 versus the non-atrophy group; ^#^*p* < 0.05 versus the MAG

### Baseline PG levels in different regions

According to regional grouping, further analysis of the OLGA-0 group showed that PGI levels were significantly different among five regions in China. There were also significant differences in PGII levels among the five regions, except for Central/Northern versus Southern China. PGR levels in Southern China were higher than those of the other regions (Table 3).

**Table 3:** Differences in baseline PG levels in different regions (OLGA-0 group)

	Total (n = 1590)	Southern China (n = 254)	Eastern China (n = 574)	Southwest China (n = 210)	Northeast China (n = 332)	Central/Northern China (n = 220)
PGI (ng/ml)	90.4 (85.2)	151.5 (124.1)^a^	63.8 (49.4)^b^	72.0 (49.2)^c^	119.7 (99.8)^d^	89.2 (82.1)^e^
PGII (ng/ml)	8.3 (11.7)	8.9 (9.8)^a^	7.4 (6.6)^b^	6.0 (5.1)^c^	11.0 (13.5)^d^	10.0 (8.0)^ae^
PGR	10.1 (16.0)	16.9 (13.4)^a^	7.9 (5.2)^b^	11.6 (7.9)^c^	11.5 (7.1)^dc^	8.0 (14.0)^be^

^a, b, c, d^^,e^*p* > 0.05 when groups share the same letter

### Analysis of the influencing factors of PG

Baseline data from five regions were further analyzed. There were significant differences in age, ethnicity, dietary habits, smoking and *Hp* infection rate among the five regions. In addition, except for the Eastern China, there is no difference in the proportion of ethnic minorities in the other four regions. The *Hp* infection rate was higher in the southwest (53.9%). People in Northeast China, Southwest China and Southern China had a higher proportion of high-salt diet (32.5%, 17.6% and 16.1%, respectively). Eastern China had the best consumption habits of fruits and vegetables (95.3% and 91.3% ate fruits and vegetables regularly, respectively). There was no difference in the consumption habits of vegetables in the other four regions. But Southern China had the least consumption habits of fruits (only 39.8%). Southwest China had the best dairy intake habit (42.9%), followed by Eastern China (31.2%), and the other three regions had no difference in dairy intake. Central/Northern China and Southern China had the highest smoking rate (42.7% and 31.4%, respectively), and Northeast China had the lowest smoking rate (11.7%) (Table 4).

**Table 4:** Differences in sex, age, smoking, dietary habits and *Hp* infection in different regions (OLGA-0 group)

	Total (n = 1590)	Southern China (n = 254)	Eastern China (n = 574)	Southwest China (n = 210)	Northeast China (n = 332)	Central/Northern China (n = 220)	*p* value
Age(years), mean (SD)	51.9(9.9)	52.9 (8.6)^a^	49.1 (9.8)^b^	49.3 (9.3)^b^	55.9 (8.5)^c^	54.6 (11.2)^ac^	
Gender							0.025
Male, n (%)	866	133 (52.4)^ab^	341 (59.4)^b^	117 (55.7)^ab^	164 (49.4)^a^	111 (50.5)^ab^	
Female, n (%)	724	121 (47.6)	233 (40.6)	93 (44.3)	168 (50.6)	109 (49.5)	
*Hp* infection							< 0.001
Positive, n (%)	583	137 (53.9)^a^	235 (40.9)^b^	69 (32.9)^bc^	97 (29.2)^cd^	45 (20.5)^d^	
Negative, n (%)	1007	117 (46.1)	339 (59.1)	141 (67.1)	235 (70.8)	175 (79.5)	
Nationality							< 0.001
Minority, n (%)	101	32 (12.6)^a^	0 (0)^b^	16 (7.6)^a^	33 (9.9)^a^	20 (9.1)^a^	
Han nationality, n (%)	1489	222 (87.4)	574 (100)	194 (92.4)	299 (90.1)	200 (90.9)	
High salt diet							< 0.001
Yes, n (%)	240	41 (16.1)^a^	30 (5.2)^b^	37 (17.6)^a^	108 (32.5)^c^	24 (10.9)^a^	
No, n (%)	1350	213 (84.9)	544 (94.8)	173 (82.4)	224 (67.5)	196 (89.1)	
Fruits							< 0.001
Frequently	1005	101 (39.8)^a^	524 (91.3)^b^	122 (58.1)^c^	189 (56.9)^c^	124 (56.4)^c^	
Occasionally	585	153 (60.2)	50 (8.7)	88 (41.9)	143 (43.1)	96 (43.6)	
Vegetables							< 0.001
Frequently	1416	208 (81.9)^a^	547 (95.3)^b^	184 (87.6)^a^	291 (87.6)^a^	186 (84.5)^a^	
Occasionally	174	46 (18.1)	27 (4.7)	26 (12.4)	41 (12.3)	34 (15.5)	
Dairy intake							< 0.001
Frequently	373	19 (7.5)^a^	179 (31.2)^b^	90 (42.9)^c^	91 (27.4)^b^	57 (25.9)^b^	
Occasionally	1217	235 (92.5)	395 (68.8)	120 (57.1)	241 (72.6)	163 (74.1)	
Smoking							< 0.001
Yes	410	80 (31.4)^a^	144 (25.1)^b^	53 (25.2)^b^	39 (11.7)^c^	94 (42.7)^a^	
No	1180	172 (68.5)	430 (74.9)	157 (74.8)	293 (88.3)	126 (57.3)	

^a,b,c,d,e^*p* > 0.05 when groups share the same letter. *p* value refers to comparison among the five regions

## Discussion

The progression from chronic non-atrophic gastritis, via atrophic gastritis (AG) and intestinal metaplasia (IM), to dysplasia, termed Correa’s cascade [15], is widely considered a common evolution path of the intestinal type of noncardia GC. AG is the turning point, and represents a precancerous lesion [16–18]. The annual incidence rates of gastric cancer were found to be 0.1%, 0.25%, 0.6%, and 6% in patients with atrophic gastritis, intestinal metaplasia, mild-to-moderate dysplasia, and severe dysplasia within 5 years after diagnosis [19]. The OLGA classification is an effective way of classifying gastritis and grading cancer risk based on histopathological findings of biopsy specimens [20, 21]. Massimo Rugge’s prospective studies explored the association between OLGA classification and the risk for gastric cancer, and confirmed that OLGA staging reliably predicts the risk for development of gastric epithelial neoplasia [22, 23]. The high-risk stage (defined as stage III or IV of the OLGA classification) is closely associated with high risk of GC [22–25]. In this study, we found 17.3% (332/1922) of the total cases were diagnosed with AG; more specifically, 2.5% (49/1922) of the cases presented with severe AG (stage III and IV of the OLGA classification), in the asymptomatic health checkup population. And the PG levels especially PGR showed significant different. Effectively screening and management of those AG patients especially severe AG patients, making full use of non-invasive testing methods such as PG in general population, has a great significance in terms of reducing the incidence of GC and improving the early detection rate.

The mean *Hp* infection rate is 39.4%, which is similar with our past research [10]. The age and *Hp* infection rate increased with the increase of the atrophy degree, which further suggested that *Hp* infection and age may be one of the causes of atrophic gastritis. Meta-analysis studies showed that the risk of gastric cancer is reduced after *Hp* eradication, and the benefit varied with or without precancerous lesion [26, 27]. Extensive testing and treatment of *Hp* in general population is effective and necessary.

Serum PG can reflect the morphological and functional status of gastric mucosa. PGI is produced by gastric fundus gland cells, and its decrease rate is related to the increase of gastric atrophy, while PGII has a greater correlation with gastric fundus mucosa lesions (relative to gastric antrum mucosa), and its increase is related to gastric fundus duct atrophy, intestinal metaplasia or pseudopylori metaplasia and dysplasia. Progressive reduction of PGR is associated with progressive gastric mucosal atrophy and gastric cancer [1]. A meta-analysis of 31 studies including 1520 patients with gastric cancer and 2265 patients with atrophic gastritis found that serum PG has great potential as a non-invasive, population-based screening tool for gastric cancer and atrophic gastritis [2]. Zoalfaghari [13] confirmed that serum PGI and PGR are potential serological markers for the screening of atrophic gastritis with high sensitivity and specificity. However, PG cutoff values varied in different studies, and the cut-off values of domestic studies were also inconsistent [5–8, 11, 12]. Our previous study found that the cutoff value for the diagnosis of atrophic gastritis was PGI ≤ 50.3 ng/ml, and there were differences between the *Hp* infected group and the non *Hp*-infected group [10]. Therefore, we conducted this study to explore whether the differences of the above cut-off values were due to the different baseline PG levels.

In this study, there were significant differences in baseline PG levels among the different regions in China. In terms of PGR, we found significant differences between Northeast and Southwest China. As shown in Tables 3 and 4, there were differences in the baseline data from different regions, especially in the *Hp* infection rate. Therefore, those differences of baseline PG levels may be related to these baseline data. Interestingly, there were significant differences in nationality, age, dietary habits, smoking, and *Hp* infection rates between Eastern China and Central/Northern China, but we found no difference in PGR levels. However, there were significant differences in PGR levels between Eastern China and Southwest China, and we found no differences in gender, smoking, age and *Hp* infection rate except for dietary habits and national differences. Above results suggests that in the follow-up studies of evaluating the PG cutoff values in China, it may be necessary to further consider the influence of ethnic groups (Southern, Northeast and Southwest China are the areas where ethnic minorities gather), dietary habits, *Hp* infection and other factors, and a larger sample and multi-center study is needed to explore the diagnostic criteria in China.

## Conclusions

In conclusion, the baseline PG levels varied in different regions of China. The influencing factors include age, nationality, dietary habits, smoking and *Hp* infection rate, and so on. In the follow-up studies of PG cut-off value, different PG cut-off values based on regions may be more effective in the screening of gastric cancer and precancerous lesions.

## Data Availability

All data generated or analyzed during this study are included in this published article and its supplementary information files.

## Abbreviations

AG: Atrophic gastritis
GC: Gastric cancer
*Hp*: *Helicobacter pylori*
IM: Intestinal metaplasia
OLGA: Operative Link on Gastritis Assessment
PG: Pepsinogen
PGI: Pepsinogen I
PGII: Pepsinogen II
PGR: PGI/PGII ratio
